# Liver fibrosis indices predict malignant cerebral edema after endovascular therapy for anterior circulation large vessel occlusion stroke: a retrospective cohort study

**DOI:** 10.3389/fneur.2025.1569698

**Published:** 2025-07-25

**Authors:** Weiwei Gao, Yanan Zhao, Jingjing She, Ziwei Wu, Lijuan Cai, Jianzhong Lin, Xingyu Chen, Renjing Zhu

**Affiliations:** ^1^Department of Neurology, Zhongshan Hospital of Xiamen University, School of Medicine, Xiamen University, Xiamen, China; ^2^Xiamen Clinical Research Center for Cerebrovascular Diseases, Xiamen, China; ^3^Xiamen Quality Control Center for Stroke, Xiamen, China; ^4^The School of Clinical Medicine, Fujian Medical University, Fuzhou, China; ^5^School of Medicine, Xiamen University, Xiamen, China; ^6^Department of MRI, Zhongshan Hospital of Xiamen University, School of Medicine, Xiamen University, Xiamen, China

**Keywords:** large vessel occlusion, malignant cerebral edema, endovascular therapy, liver fibrosis indices, anterior circulation

## Abstract

**Objective:**

To investigate the association between liver fibrosis indices and malignant cerebral edema (MCE) following endovascular therapy (EVT) for anterior circulation large vessel occlusion (LVO) stroke.

**Methods:**

This single-center, retrospective cohort study consecutively enrolled 340 anterior circulation LVO stroke patients who underwent EVT between January 2018 and December 2024. The primary outcome was MCE, defined as a midline shift >5 mm at the level of the septum pellucidum or pineal gland, accompanied by the disappearance of the perimesencephalic cistern or the need for decompressive craniectomy. Multivariable logistic regression models were used to assess the independent associations of eight liver fibrosis indices with MCE, and restricted cubic spline regression analysis (RCS) was employed to explore their nonlinear relationships. The predictive performance was evaluated using receiver operating characteristic curves.

**Results:**

MCE occurred in 69 patients (20.3%). After adjusting for multiple confounders, elevated fibrosis-4 index (FIB-4) (OR = 1.41, 95% CI: 1.09–1.82), modified FIB-4 (OR = 1.14, 95% CI: 1.05–1.23), aspartate aminotransferase (AST)/alanine aminotransferase (ALT) ratio (ARR) (OR = 2.35, 95% CI: 1.57–3.52), and AST/ALT-platelet ratio index (AARPRI) (OR = 2.63, 95% CI: 1.69–4.10) were independently associated with an increased risk of MCE, while FIB-5 index showed a negative association (OR = 0.89, 95% CI: 0.83–0.94). These liver fibrosis indices demonstrated moderate predictive performance (AUC: 0.65–0.68). RCS analysis revealed that most liver fibrosis indices exhibited an overall increasing dose–response relationship with MCE risk (both P-overall<0.05).

**Conclusion:**

Non-invasive liver fibrosis indices could serve as novel biomarkers for risk stratification of MCE following EVT for stroke. These readily available tools may help identify high-risk patients who might benefit from early preventive interventions.

## 1 Introduction

Stroke is a leading cause of mortality and disability worldwide, with acute ischemic stroke (AIS) accounting for approximately 62.4% of all cases (1, 2). Large vessel occlusion (LVO) is a distinct subtype of AIS, with an incidence ranging from 28 to 46% (3). Patients with LVO have attracted significant clinical attention due to their rapid disease progression, severe neurological deficits, and poor prognosis (4). Endovascular therapy (EVT) has significantly improved the functional outcomes of LVO patients by achieving rapid vessel recanalization and salvaging the ischemic penumbra, becoming the standard treatment strategy in the hyperacute phase (5). However, in real-world settings, a considerable proportion of patients still experience unfavorable outcomes despite successful recanalization of the occluded vessel. Among these complications, malignant cerebral edema (MCE) is one of the most severe. A recent secondary analysis of a multicenter randomized trial showed that approximately 40% of patients developed post-procedural midline shift caused by cerebral edema after EVT (6). Alarmingly, another latest study involving 33 stroke centers found that MCE accounted for 50.6% of deaths within 30 days after EVT (7). Although early decompressive craniectomy can significantly reduce mortality and disability in MCE patients (8), early prediction and prevention of this lethal complication remain critical issues to be addressed.

Non-alcoholic fatty liver disease (NAFLD) is the most common chronic liver disease globally, with a prevalence of 30.1% (9). Among NAFLD patients, liver fibrosis is considered a key stage. Recent evidence has highlighted the potential role of liver fibrosis in the pathophysiology of stroke, possibly affecting stroke outcomes through multiple mechanisms such as endothelial dysfunction, systemic inflammation, and oxidative stress (10–13). Previous studies have demonstrated that liver fibrosis is associated with more severe neurological deficits, increased in-hospital mortality, poor long-term functional outcomes, and an elevated risk of recurrent stroke in AIS patients (14–17). Furthermore, an elevated fibrosis-4 (FIB-4) index has been identified as an independent predictor of hemorrhagic transformation and symptomatic intracerebral hemorrhage after intravenous thrombolysis and EVT (18–21). However, the relationship between liver fibrosis and MCE following EVT has not yet been explored. Moreover, existing studies have primarily relied on a single fibrosis index, which may limit the accurate assessment of liver fibrosis severity.

To address this knowledge gap, we evaluated the associations of eight non-invasive liver fibrosis indices with MCE in anterior circulation LVO stroke patients undergoing EVT. This study aimed to establish the potential value of liver fibrosis markers in risk stratification for MCE after EVT and provide a scientific basis for developing targeted prevention strategies.

## 2 Manuscript formatting

### 2.1 Study design and population

This study was a single-center, retrospective cohort study based on a prospectively maintained electronic medical record database. The study protocol was approved by the medical ethics committee, and the requirement for informed consent was waived due to the retrospective nature of the study. We established an inception cohort comprising all consecutive anterior circulation LVO-AIS patients who underwent EVT at our comprehensive stroke center between January 1, 2018, and December 31, 2024. The inception point was defined as the time of hospital admission for acute stroke, with patients followed until hospital discharge, death, or development of MCE, whichever occurred first. All enrolled patients had anterior circulation LVO confirmed by preoperative computed tomography angiography, magnetic resonance angiography, or digital subtraction angiography (DSA), including the terminal internal carotid artery or the M1/M2 segment of the middle cerebral artery.

The exclusion criteria were as follows: (1) age < 18 years; (2) presence of intracranial hemorrhage on baseline head computed tomography (CT) or magnetic resonance imaging; (3) pre-stroke modified Rankin Scale score > 2; (4) history of severe liver diseases (including viral hepatitis, cirrhosis, or primary liver cancer) or current use of potentially hepatotoxic drugs; (5) severe renal insufficiency; and (6) incomplete clinical laboratory or imaging data. Considering the characteristics of real-world studies, we did not exclude patients with a history of chronic alcohol consumption (21).

### 2.2 Data collection and clinical assessment

Two trained researchers independently extracted data using a standardized electronic spreadsheet, and any discrepancies were resolved through discussion with a senior researcher. Baseline characteristics included demographic characteristics (age and sex), cerebrovascular risk factors (Current smoker, alcohol consumption, hypertension, diabetes, hyperlipidemia, atrial fibrillation, previous stroke or transient ischemic attack, and coronary heart disease), and vital signs at admission (systolic and diastolic blood pressure).

### 2.3 Procedural characteristics

Procedural parameters were recorded in real-time by the operators, including intravenous thrombolysis status, key time points (onset-to-puncture time, onset-to-reperfusion time, and puncture-to-reperfusion time), and procedural details (number of mechanical thrombectomy attempts, device strategy [stent retriever, aspiration catheter, or combined use], and balloon angioplasty). The modified Thrombolysis in Cerebral Infarction (mTICI) grading system was used to assess the reperfusion status, which was determined based on the final DSA results. Successful reperfusion was defined as mTICI grades 2b − 3.

### 2.4 Laboratory analysis and liver fibrosis indices

All patients had fasting venous blood samples collected within 24 h of admission, which were analyzed by the hospital’s central laboratory using standardized procedures. The laboratory tests included total protein, albumin, total bilirubin, aspartate aminotransferase (AST), alanine aminotransferase, *γ*-glutamyl transferase, alkaline phosphatase, lipid profile (total cholesterol, triglycerides, high-density lipoprotein cholesterol, low-density lipoprotein cholesterol), fasting blood glucose (FBG), blood urea nitrogen (BUN), creatinine, uric acid (UA), and platelet count (PLT).

**Figure 1:**
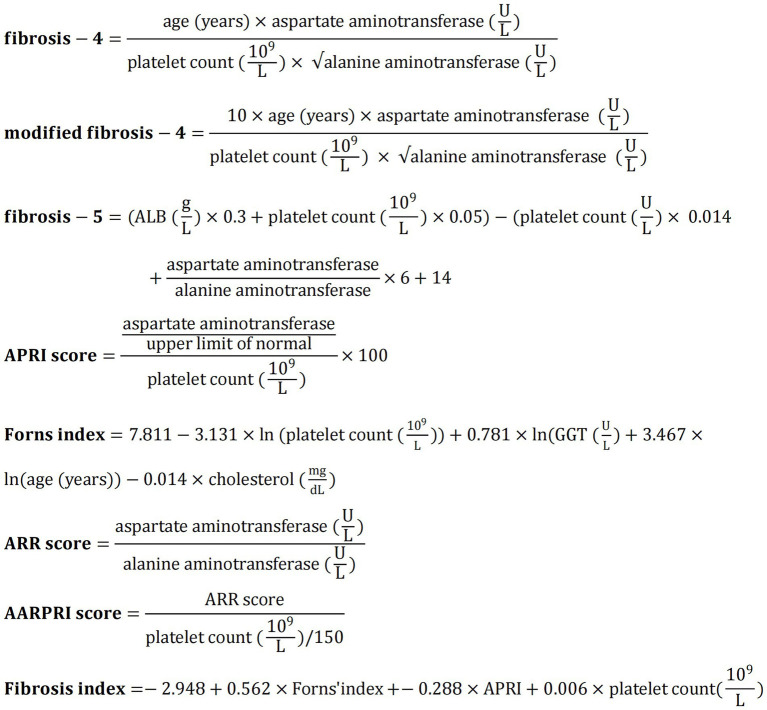
Calculation formulas for eight non-invasive liver fibrosis indices. APRI, aspartate aminotransferase to platelet ratio index; GGT, *γ*-glutamyl transferase; ARR, aspartate aminotransferase/alanine aminotransferase ratio; AARPRI, AAR-platelet count ratio index.

Eight validated non-invasive liver fibrosis indices were calculated (19): FIB-4 index, modified FIB-4 index (mFIB-4), fibrosis-5 index (FIB-5), AST to platelet ratio index (APRI), Forns index, AST/ALT ratio (ARR), AST/ALT-PLT ratio index (AARPRI), and liver fibrosis index (LFI). The specific calculation formulas are shown in Figure 1.

### 2.5 Outcome measures

The primary outcome of this study was MCE, defined as a midline shift exceeding 5 mm at the level of the septum pellucidum or pineal gland, accompanied by the disappearance of the perimesencephalic cistern or the need for decompressive craniectomy (22–24). To systematically monitor the occurrence and progression of cerebral edema, all patients underwent head CT examinations according to a standardized protocol, including immediately after endovascular treatment, at 24 h, and at 72 h. Additional CT scans were performed immediately upon neurological deterioration or signs of edema progression on previous imaging. For patients diagnosed with MCE, imaging assessments were conducted every 12–24 h to closely monitor the progression of cerebral edema.

To ensure the objectivity and accuracy of the assessments, all imaging evaluations were performed using a double-blind method by two senior neuroradiologists and one attending neurologist independently. The assessors were blinded to all clinical data of the patients, and any discrepancies were resolved through discussion by an expert panel.

### 2.6 Statistical analysis

All statistical analyses were performed using R software (Version 4.2.2). The Shapiro–Wilk test was used to assess the normality of continuous variables. Normally distributed continuous variables were expressed as mean ± standard deviation and compared between groups using independent samples t-tests. Non-normally distributed continuous variables were expressed as median [interquartile range (IQR)] and compared using the Mann–Whitney U test. Categorical variables were expressed as numbers (percentages) and compared using the chi-square test or Fisher’s exact test.

To evaluate the predictive value of liver fibrosis indices for MCE, we constructed receiver operating characteristic (ROC) curves and calculated the area under the curve (AUC) and its 95% confidence interval. The Youden index method was used to determine the optimal diagnostic cut-off values, and the corresponding sensitivity and specificity were calculated. Based on the variables that were statistically significant (*p* < 0.05) in the univariate analysis, three stepwise-adjusted multivariable logistic regression models were constructed to explore the associations between liver fibrosis indices and the outcome. Model 1 was the unadjusted baseline model; Model 2 adjusted for clinical factors (smoking status, atrial fibrillation, coronary heart disease, baseline NIHSS score, GCS score, ASPECTS score, puncture-to-reperfusion time, number of mechanical thrombectomy attempts, and successful reperfusion status); Model 3 further adjusted for laboratory parameters (aspartate aminotransferase, fasting blood glucose, blood urea nitrogen, uric acid, and platelet count).

Restricted cubic spline (RCS) regression models were used to assess potential nonlinear associations, with knots set at the 5th, 35th, 65th, and 95th percentiles, and the overall association and nonlinear component were tested separately. To evaluate the robustness of the study results, we performed sensitivity analyses, including bootstrap validation (1,000 repetitions) and stratified analyses based on key baseline characteristics. All statistical tests were two-sided, and *p* < 0.05 was considered statistically significant.

## 3 Results

### 3.1 Patient characteristics

A total of 340 anterior circulation LVO-AIS patients were enrolled in this study (Table 1), of whom 69 (20.3%) developed MCE. The median age of all patients was 67 years (IQR: 57–76 years), and 225 (66.18%) were male. Compared to the non-MCE group, patients in the MCE group had a lower proportion of smokers (23.19% vs. 42.07%, *p* = 0.004) but exhibited a higher incidence of atrial fibrillation (55.07% vs. 40.59%, *p* = 0.030) and coronary heart disease (20.29% vs. 11.07%, *p* = 0.042). Patients in the MCE group had more severe neurological deficits at admission, as evidenced by a higher median NIHSS score (17 vs. 14, *p* < 0.001) and a lower median GCS score (11 vs. 13, *p* < 0.001). Additionally, the baseline ASPECTS score was significantly lower in the MCE group (8 vs. 9, *p* = 0.031). Regarding procedural characteristics, the MCE group demonstrated a longer puncture-to-reperfusion time (median 82 min vs. 75 min, *p* = 0.015) and more mechanical thrombectomy attempts [2 (1–3) vs. 2 (1, 2), *p* < 0.001]. Notably, the successful reperfusion rate was significantly lower in the MCE group than in the non-MCE group (78.26% vs. 87.82%, *p* = 0.042).

**Table 1:** Baseline characteristics of patients with anterior circulation large vessel occlusion stroke according to MCE status.

Variables	Overall (*n* = 340)	Non-MCE (*n* = 271)	MCE (*n* = 69)	*p* value
Demographics				
Age, years, median (IQR)	67 (57, 76)	66 (57, 76)	69 (56, 76)	0.788
Male sex, *n* (%)	225 (66.18)	180 (66.42)	45 (65.22)	0.850
Current smoker, *n* (%)	130 (38.24)	114 (42.07)	16 (23.19)	0.004^*^
Alcohol consumption, *n* (%)	88 (25.88)	68 (25.09)	20 (28.99)	0.510
Medical history				
Hypertension, *n* (%)	226 (66.47)	181 (66.79)	45 (65.22)	0.805
Diabetes mellitus, *n* (%)	97 (28.53)	74 (27.31)	23 (33.33)	0.322
Hyperlipidemia, *n* (%)	79 (23.24)	65 (23.99)	14 (20.29)	0.516
Atrial fibrillation, *n* (%)	148 (43.53)	110 (40.59)	38 (55.07)	0.030^*^
Previous stroke/TIA, *n* (%)	54 (15.88)	48 (17.71)	6 (8.70)	0.067
Coronary artery disease, *n* (%)	44 (12.94)	30 (11.07)	14 (20.29)	0.042^*^
Clinical presentation				
SBP, mmHg, median (IQR)	148 (132, 163)	149 (133, 163)	144 (131, 162)	0.427
DPB, mmHg, median (IQR)	87 (77, 97)	87 (76, 98)	87 (77, 93)	0.650
Baseline NIHSS score, median (IQR)	15 (12, 19)	14 (11, 18)	17 (15, 21)	<0.001^*^
Baseline GCS score, median (IQR)	12 (10, 14)	13 (10, 15)	11 (9, 13)	<0.001^*^
Baseline ASPECT score, median (IQR)	9 (8, 10)	9 (8, 10)	8 (6, 10)	0.031^*^
Stroke etiology, *n* (%)				0.071
Large-artery atherosclerosis	162 (47.65)	134 (49.45)	28 (40.58)	
Cardioembolism	160 (47.06)	120 (44.28)	40 (57.97)	
Other	18 (5.29)	17 (6.27)	1 (1.45)	
Procedural characteristics				
Intravenous thrombolysis, *n* (%)	146 (42.94)	116 (42.80)	30 (43.48)	0.920
OPT, min, median (IQR)	373 (266, 555)	370 (260, 560)	389 (305, 521)	0.454
PRT, min, median (IQR)	76 (48, 99)	75 (45, 97)	82 (60, 120)	0.015^*^
ORT, min, median (IQR)	460 (338, 655)	457 (322, 660)	463 (373, 630)	0.430
NOTA, median (IQR)	2.00 (1.00, 2.00)	2.00 (1.00, 2.00)	2.00 (1.00, 3.00)	<0.001^*^
Successful reperfusion, n (%)	292 (85.88)	238 (87.82)	54 (78.26)	0.042^*^
Treatment strategy, *n* (%)				0.698
Stent retriever	72 (21.18)	54 (19.93)	18 (26.09)	
Aspiration	21 (6.18)	18 (6.64)	3 (4.35)	
Combined approach	227 (66.76)	183 (67.53)	44 (63.77)	
Balloon angioplasty, *n* (%)	62 (18.24)	51 (18.82)	11 (15.94)	0.581

Values are presented as median (IQR) or number (percentage). *p < 0.05 was considered statistically significant. MCE, malignant cerebral edema; IQR, interquartile range; TIA, transient ischemic attack; SBP, systolic blood pressure; DBP, diastolic blood pressure; NIHSS, National Institutes of Health Stroke Scale; GCS, Glasgow Coma Scale; ASPECTS, Alberta Stroke Program Early Computed Tomography Score; OPT, onset-to-puncture time; PRT, puncture-to-reperfusion time; ORT, onset-to-recanalization time; NOTA, number of thrombectomy attempts.

### 3.2 Laboratory characteristics of patients

Laboratory tests revealed significant differences between the MCE and non-MCE groups (Table 2). The MCE group exhibited significantly elevated levels of AST (*p* = 0.003), FBG (*p* < 0.001), urea (*p* = 0.050), and UA (*p* = 0.030). In contrast, the PLT count was significantly lower in the MCE group (*p* = 0.001). The assessment of liver fibrosis indices showed that the MCE group had significantly higher FIB-4 (*p* < 0.001), mFIB-4 (*p* < 0.001), APRI (*p* < 0.001), ARR (*p* < 0.001), and AARPRI (*p* < 0.001) scores. Conversely, the FIB-5 index was significantly lower in the MCE group (*p* < 0.001). The differences in the Forns index and LFI between the two groups did not reach statistical significance.

**Table 2:** Laboratory parameters and liver fibrosis indices in patients with and without MCE.

Variables	Overall (*n* = 340)	Non-MCE (*n* = 271)	MCE (*n* = 69)	*p* value
Total protein, g/L, median (IQR)	65 (62, 70)	65 (62, 69)	64 (60, 70)	0.105
Albumin, g/L, median (IQR)	37.2 (34.6, 39.6)	37.2 (35.0, 39.5)	37.3 (33.4, 39.7)	0.590
Total bilirubin, μmol/L, median (IQR)	16 (13, 21)	16 (12, 20)	17 (13, 23)	0.113
ALT, U/L, median (IQR)	16 (12, 24)	17 (12, 24)	15 (11, 21)	0.133
AST, U/L, median (IQR)	24 (19, 31)	24 (19, 30)	27 (22, 38)	0.003^*^
GGT, U/L, median (IQR)	28 (19, 46)	29 (20, 45)	26 (19, 50)	0.589
ALP, U/L, median (IQR)	77 (65, 93)	78 (66, 95)	74 (62, 87)	0.115
Triglycerides, mmol/L, median (IQR)	1.06 (0.78, 1.43)	1.08 (0.81, 1.42)	0.99 (0.70, 1.46)	0.137
Total cholesterol, mmol/L, median (IQR)	4.34 (3.59, 5.09)	4.35 (3.64, 5.10)	4.30 (3.12, 5.01)	0.181
HDL-C, mmol/L, median (IQR)	1.17 (1.00, 1.34)	1.18 (1.01, 1.34)	1.12 (0.97, 1.34)	0.169
LDL-C, mmol/L, median (IQR)	2.85 (2.23, 3.41)	2.86 (2.30, 3.44)	2.83 (1.85, 3.22)	0.173
FBG, mmol/L, median (IQR)	7.5 (6.2, 9.7)	7.2 (6.0, 9.2)	8.8 (7.5, 12.0)	<0.001^*^
BUN, mmol/L, median (IQR)	5.46 (4.10, 7.20)	5.40 (4.05, 6.95)	5.80 (4.30, 8.80)	0.050^*^
Creatinine, μmol/L, median (IQR)	72 (59, 87)	72 (58, 85)	74 (60, 117)	0.052
Uric acid, μmol/L, median (IQR)	329 (270, 415)	324 (266, 406)	367 (285, 460)	0.030^*^
Platelet count, ×10^9^/L, median (IQR)	202 (168, 238)	207 (176, 243)	180 (150, 210)	0.001
FIB-4, median (IQR)	2.00 (1.34, 3.01)	1.86 (1.32, 2.75)	2.97 (1.48, 4.39)	<0.001
Modified FIB-4, median (IQR)	4.8 (3.0, 7.4)	4.5 (2.9, 6.7)	7.0 (3.7, 10.8)	<0.001
FIB-5, median (IQR)	-3 (−7, 1)	-2 (−6, 1)	-7 (−12, −1)	<0.001
APRI score, median (IQR)	0.34 (0.24, 0.48)	0.33 (0.23, 0.44)	0.46 (0.29, 0.69)	<0.001
Forns index, mean (SD)	6.00 ± 1.67	5.91 ± 1.66	6.35 ± 1.67	0.059
ARR score, median (IQR)	1.48 (1.08, 2.04)	1.39 (1.06, 1.85)	1.92 (1.35, 2.52)	<0.001
AARPRI, median (IQR)	1.10 (0.77, 1.55)	1.06 (0.74, 1.43)	1.61 (0.94, 2.52)	<0.001
Fibrosis index, mean (SD)	1.80 ± 0.76	1.77 ± 0.76	1.92 ± 0.75	0.133

*p < 0.05 was considered statistically significant. MCE, malignant cerebral edema; IQR, interquartile range; SD, standard deviation; ALT, alanine aminotransferase; AST, aspartate aminotransferase; GGT, γ-Glutamyl transferase; FBG, fasting blood glucose; BUN, blood urea nitrogen; HDL-C, high-density lipoprotein cholesterol; LDL-C, low-density lipoprotein cholesterol; FIB-4, fibrosis-4 index; FIB-5, fibrosis-5 index; APRI, aspartate aminotransferase to platelet ratio index; ARR, aspartate aminotransferase to alanine aminotransferase ratio; AARPRI, AST/ALT-platelet ratio index.

### 3.3 Predictive value of liver fibrosis indices

**Figure 2:**
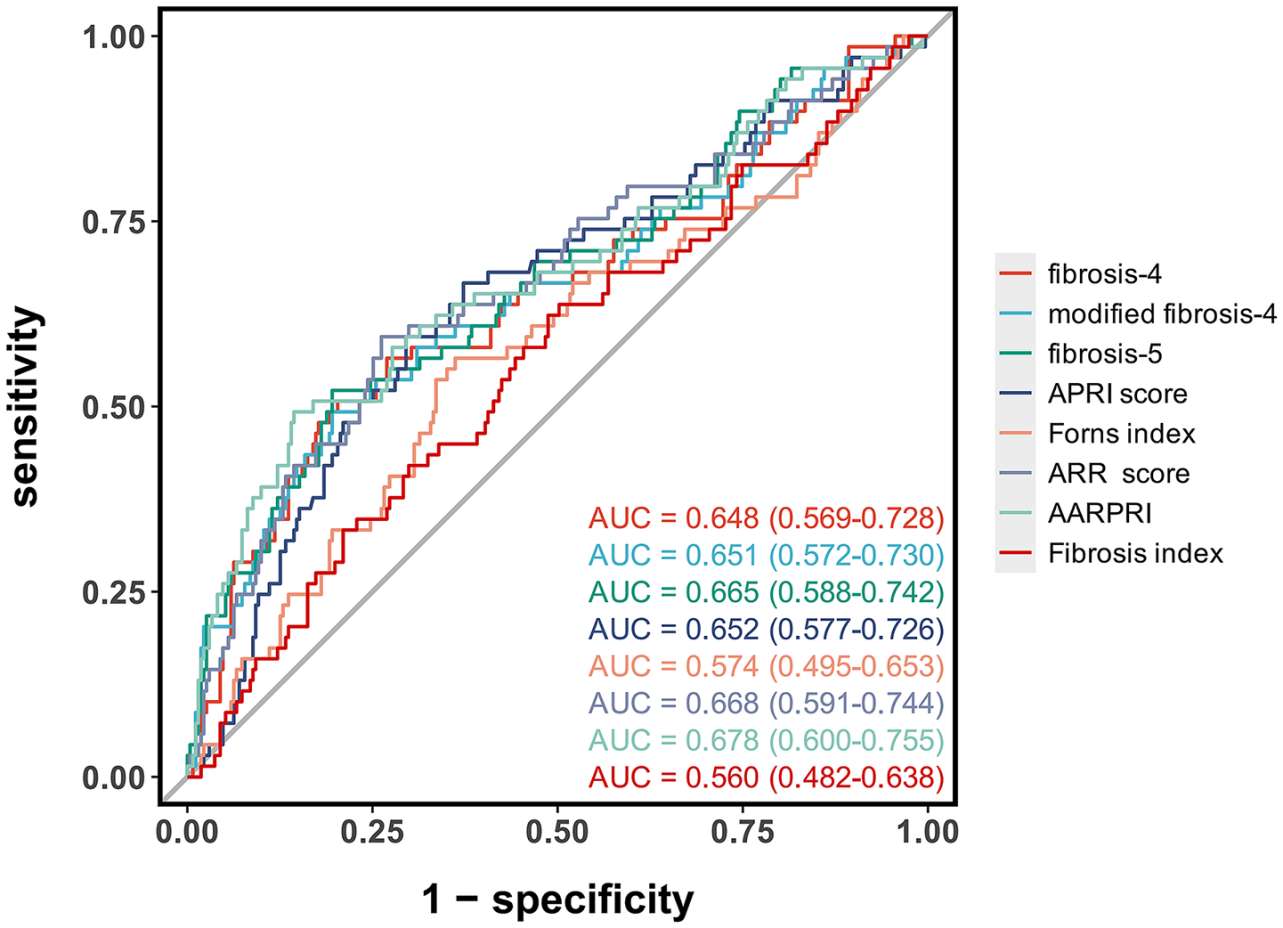
Receiver operating characteristic curves of liver fibrosis indices for predicting malignant cerebral edema after endovascular therapy. The area under the curve (AUC) values with 95% confidence intervals are shown for each liver fibrosis index.

ROC curve analysis showed that AARPRI had the highest predictive value for MCE after EVT (AUC = 0.68, 95% CI: 0.60–0.76), outperforming other liver fibrosis indices (Figure 2; Table 3). When the optimal cut-off value for AARPRI was set at 1.73, the diagnostic accuracy of AARPRI was 0.78 (95% CI: 0.73–0.83), with high sensitivity (0.86, 95% CI: 0.81–0.90) but moderate specificity (0.49, 95% CI: 0.37–0.61). The ARR and FIB-5 indices demonstrated comparable discriminatory performance (AUC = 0.67, 95% CI: 0.59–0.75). The AUCs for FIB-4, mFIB-4, and APRI were all 0.65, with FIB-4 exhibiting significant sensitivity (0.80, 95% CI: 0.75–0.84) and accuracy (0.74, 95% CI: 0.69–0.78) at a cut-off value of 2.97.

**Table 3:** Diagnostic performance of liver fibrosis indices for predicting MCE after endovascular therapy.

Parameter	AUC (95% CI)	Accuracy (95% CI)	Sensitivity (95% CI)	Specificity (95% CI)	Cut-off value
fibrosis-4	0.65 (0.57–0.73)	0.74 (0.69–0.78)	0.80 (0.75–0.84)	0.51 (0.39–0.63)	2.97
modified fibrosis-4	0.65 (0.57–0.73)	0.74 (0.69–0.79)	0.80 (0.76–0.85)	0.49 (0.37–0.61)	7.35
fibrosis-5	0.67 (0.59–0.74)	0.25 (0.21–0.30)	0.20 (0.15–0.24)	0.48 (0.36–0.60)	−6.67
APRI score	0.65 (0.58–0.73)	0.68 (0.62–0.73)	0.70 (0.64–0.75)	0.59 (0.48–0.71)	0.41
Forns index	0.57 (0.49–0.65)	0.62 (0.57–0.68)	0.64 (0.58–0.70)	0.57 (0.45–0.68)	6.26
ARR score	0.67 (0.59–0.74)	0.71 (0.66–0.76)	0.74 (0.69–0.79)	0.59 (0.48–0.71)	1.81
AARPRI	0.68 (0.60–0.76)	0.78 (0.73–0.83)	0.86 (0.81–0.90)	0.49 (0.37–0.61)	1.73
Fibrosis index	0.56 (0.48–0.64)	0.54 (0.48–0.59)	0.51 (0.45–0.57)	0.62 (0.51–0.74)	1.74

MCE, malignant cerebral edema; AUC, area under the receiver operating characteristic curve; CI, confidence interval; FIB-4, fibrosis-4 index; FIB-5, fibrosis-5 index; APRI, aspartate aminotransferase to platelet ratio index; ARR, aspartate aminotransferase to alanine aminotransferase ratio; AARPRI, aspartate aminotransferase to alanine aminotransferase-platelet ratio index.

### 3.4 Multivariable analysis

In the multivariable logistic regression models (Table 4), several liver fibrosis indices showed independent associations with the risk of MCE. In the unadjusted baseline model (Model 1), FIB-4, mFIB-4, FIB-5, ARR, and AARPRI were significantly associated with the risk of MCE. After further adjusting for clinical factors (Model 2), these associations remained significant. In the fully adjusted model (Model 3), each unit increase in AARPRI, ARR, FIB-4, and mFIB-4 was associated with a 163% (OR = 2.63, 95% CI: 1.69–4.10, *p* < 0.001), 135% (OR = 2.35, 95% CI: 1.57–3.52, *p* < 0.001), 41% (OR = 1.41, 95% CI: 1.09–1.82, *p* = 0.009), and 14% (OR = 1.14, 95% CI: 1.05–1.23, *p* = 0.001) increased risk of MCE, respectively. Notably, the FIB-5 index was negatively associated with the risk of MCE [OR = 0.89, 95% CI: 0.83–0.94, *p* < 0.001]. In contrast, the APRI, Forns index, and LFI did not show statistical significance in the fully adjusted model (all *p* > 0.05).

**Table 4:** Multivariable analysis of liver fibrosis indices for predicting MCE.

Variables	Model 1		Model 2		Model 3	
	OR (95% CI)	*p* value	OR (95% CI)	p value	OR (95% CI)	*p* value
FIB-4	1.30 (1.13–1.51)	<0.001	1.32 (1.12–1.55)	<0.001	1.41 (1.09–1.82)	0.009
Modified FIB-4	1.12 (1.06–1.19)	<0.001	1.13 (1.06–1.21)	<0.001	1.14 (1.05–1.23)	0.001
FIB-5	0.90 (0.86–0.94)	<0.001	0.89 (0.85–0.94)	<0.001	0.89 (0.83–0.94)	<0.001
APRI score	1.64 (0.93–2.89)	0.086	1.78 (0.95–3.34)	0.072	6.58 (0.46–94.19)	0.165
Forns index	1.17 (1.00–1.38)	0.056	1.07 (0.87–1.31)	0.536	0.80 (0.59–1.09)	0.163
ARR score	2.09 (1.52–2.87)	<0.001	2.36 (1.63–3.43)	<0.001	2.35 (1.57–3.52)	<0.001
AARPRI	2.13 (1.57–2.89)	<0.001	2.31 (1.63–3.26)	<0.001	2.63 (1.69–4.10)	<0.001
Fibrosis index	1.31 (0.92–1.86)	0.134	1.03 (0.66–1.63)	0.884	0.70 (0.40–1.20)	0.192

MCE, malignant cerebral edema; ALT, Alanine aminotransferase; AST, aspartate aminotransferase; FIB-4, fibrosis-4; FIB-5, fibrosis-5; APRI, aspartame aminotransferase to platelet ratio index; ARR, ALT/AST ratio; AARPR, AST/ALT ratio-platelet ratio.

### 3.5 Nonlinear association analysis

**Figure 3:**
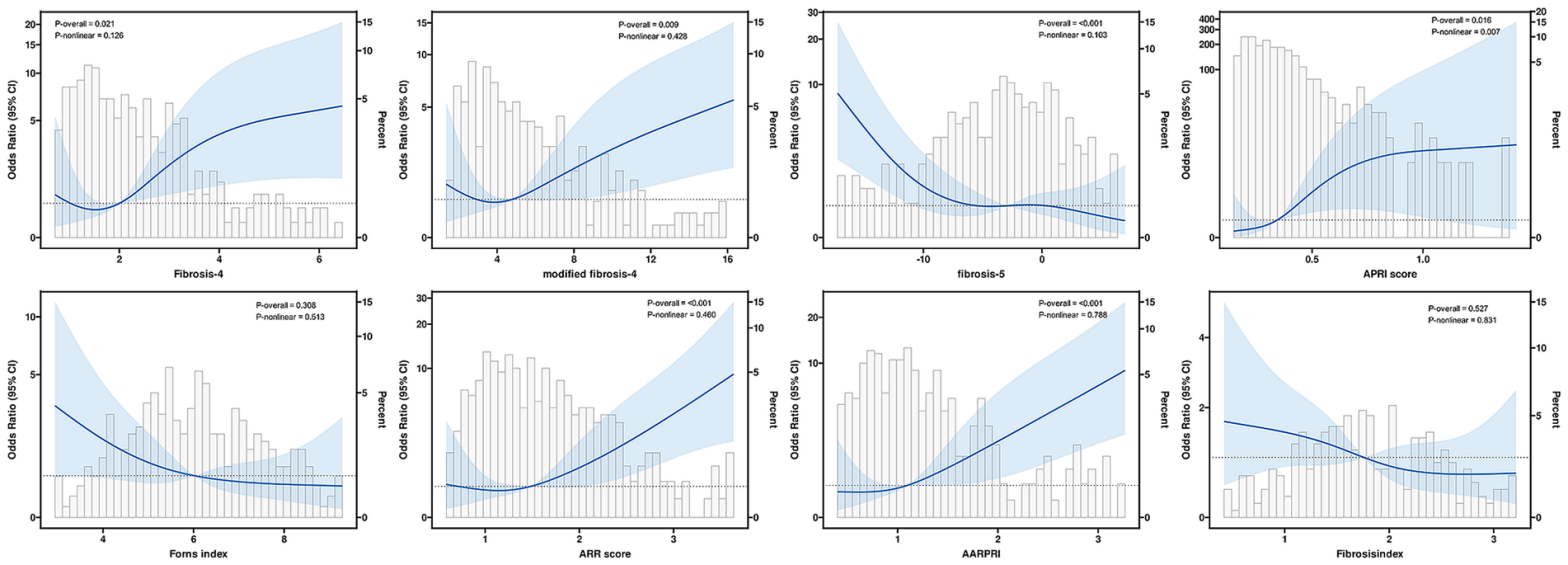
Restricted cubic spline regression analysis of the associations between liver fibrosis indices and the risk of malignant cerebral edema after endovascular therapy. The solid lines represent the odds ratios, and the shaded areas represent the 95% confidence intervals. The P-overall and P-nonlinear values are shown for each liver fibrosis index.

RCS analysis (Figure 3) showed that FIB-4 (P-nonlinear = 0.126), mFIB-4 (P-nonlinear = 0.428), APRI (P-nonlinear = 0.007), ARR (P-nonlinear = 0.460), and AARPRI (P-nonlinear = 0.786) exhibited an overall increasing dose–response relationship with the risk of MCE (both P-overall<0.05). In contrast, the FIB-5 index demonstrated a significant nonlinear negative association with the risk of MCE (P-overall<0.001, P-nonlinear = 0.103). The associations of the Forns index and LFI with the risk of MCE did not reach statistical significance (P-overall = 0.308 and 0.527, respectively) and showed no evident nonlinear trends (P-nonlinear = 0.513; P-nonlinear = 0.831).

## 4 Discussion

In this retrospective cohort study, we systematically evaluated the associations between eight non-invasive liver fibrosis indices and the risk of MCE in LVO-AIS patients undergoing EVT for the first time. Multivariable analysis demonstrated that, after adjusting for multiple confounding factors, several liver fibrosis indices (FIB-4, mFIB-4, FIB-5, ARR score, and AARPRI score) remained independently associated with the risk of MCE following EVT. RCS analysis revealed an overall increasing dose–response relationship between these indices and the risk of MCE. The predictive values of these liver fibrosis indices were similar, but AARPRI slightly outperformed the other indices. Beyond liver fibrosis indices, univariate analysis identified several additional significant associations with MCE risk. Concurrent atrial fibrillation, history of coronary heart disease, higher baseline NIHSS scores, lower GCS scores, reduced ASPECTS scores, and increased number of mechanical thrombectomy attempts were significantly associated with elevated MCE risk. Conversely, successful vessel recanalization and smoking history demonstrated protective effects. These results corroborate findings from previous studies and provide additional validation of established risk factors.

A growing body of evidence suggests that liver fibrosis is closely related to the development of AIS. Previous studies have demonstrated that the degree of liver fibrosis, based on non-invasive assessments, is associated with more severe neurological deficits at admission, higher in-hospital mortality, poorer long-term functional recovery, and an increased risk of recurrent stroke in AIS patients (14–17). Additionally, research has found that liver fibrosis indices are equally valuable in predicting complications related to acute reperfusion therapy for stroke. For example, the FIB-4 index has been shown to be an independent risk factor for hemorrhagic transformation and symptomatic intracerebral hemorrhage after intravenous thrombolysis (18, 19). Currently, studies on the relationship between liver fibrosis and the prognosis of LVO-AIS patients remain limited. Höfler et al. (21) were the first to reveal that elevated FIB-4 levels were significantly associated with unfavorable functional outcomes (mRS ≥ 3, OR = 2.15, 95% CI: 1.21–3.83, *p* = 0.009) and mortality risk (OR = 2.16, 95% CI: 1.16–4.03, *p* = 0.01) at 3 months after EVT in LVO patients. Subsequently, Xu et al. (20) further confirmed that elevated FIB-4 levels were an independent risk factor for sICH after EVT (OR = 1.306, 95% CI: 1.127–1.512, *p* = 0.001). However, prior to this study, no research had explored the relationship between liver fibrosis and MCE, a critical complication. Our study not only fills this gap but also provides a more comprehensive assessment strategy for clinical risk stratification by evaluating and comparing the predictive values of multiple liver fibrosis indices.

The pathophysiological process of MCE involves two interrelated mechanisms: cytotoxic edema and vasogenic edema (25). In the early stage of ischemic injury, energy metabolism disorders lead to the inhibition of sodium-potassium ATPase (Na^+^-K^+^-ATPase) function, causing continuous accumulation of intracellular Na^+^ in neurons and glial cells. This process disrupts the ionic balance between the intracellular and extracellular compartments, triggering cell depolarization and osmotic changes, ultimately resulting in cytotoxic cellular edema. Simultaneously, ischemic stimuli induce the upregulation of various mediators (such as thrombin, vascular endothelial growth factor, and nitric oxide synthase), which synergistically disrupt the integrity of the blood–brain barrier (26). Within 1–5 days after stroke onset, persistent blood–brain barrier dysfunction leads to plasma protein extravasation and increased interstitial osmotic pressure, exacerbating interstitial edema. Due to the anatomical characteristics of fixed intracranial volume, these pathological changes cause an increase in intracranial pressure, leading to reduced cerebral blood flow and impaired cerebral autoregulation, forming a vicious cycle that may ultimately progress to fatal brain herniation.

The increased risk of MCE after EVT in patients with liver fibrosis may involve multiple mechanisms. First, liver fibrosis can promote the occurrence of MCE by exacerbating endothelial dysfunction. Studies have found that hepatocytes in NAFLD patients can release specific circulating extracellular vesicles, which can activate vascular endothelial inflammatory responses through signaling pathways such as miRNA-novel−7 and microRNA-1. This not only promotes atherosclerotic plaque instability but also increases microvascular permeability (10, 27–29). This endothelial dysfunction may lead to impaired cerebral microvascular barrier function, creating conditions for the development of MCE.

Second, liver fibrosis may promote the progression of MCE by aggravating inflammatory responses. Du Plessis et al.’s study showed that the histological severity of NAFLD was significantly positively correlated with inflammatory mediator levels (11). Particularly in patients with non-alcoholic steatohepatitis, serum matrix metalloproteinase-9 (MMP-9) levels were significantly higher than those in the control group (12.6 ± 6.5 vs. 8.1 ± 3.5 μg/L, *p* < 0.01) (12). Evidence suggests that serum MMP-9 levels exceeding 140 μg/L can predict an increased risk of cerebral edema and poor prognosis in AIS patients (30). Moreover, in acute ischemic events, patients with liver fibrosis exhibit enhanced peripheral immune cell mobilization and tissue infiltration, which may further damage the integrity of the blood–brain barrier by amplifying the inflammatory cascade (31). Third, oxidative stress, as a common pathological basis for liver fibrosis and cardiovascular diseases, may play a key role in the occurrence of MCE. Studies have shown that oxidative stress can directly damage the structural integrity of the blood–brain barrier by degrading extracellular matrix components and disrupting tight junction proteins (13). This mechanism may explain the increased susceptibility to ischemic injury in patients with liver fibrosis.

Furthermore, the univariate findings described above corroborate previous research results. In patients with atrial fibrillation, emboli tend to cause proximal large vessel occlusion, leading to large infarcts and a higher risk of hemorrhagic transformation, thereby increasing the risk of cerebral edema formation (32, 33). The impact of coronary heart disease may be mediated through heart failure and neuroendocrine disorders. Research shows that approximately 25% of patients with ST-segment elevation myocardial infarction progress to heart failure (34), which is closely related to fatal cerebral edema (35). It is particularly noteworthy that some patients with coronary heart disease still have significantly elevated N-terminal pro-brain natriuretic peptide (NT-proBNP) levels 1 year after onset (36), and NT-proBNP has been proven to be an independent predictor of MCE after EVT (37). NT-proBNP may exacerbate cerebral edema by increasing microvascular permeability through its active product BNP, promoting albumin extravasation, and inhibiting interstitial fluid reabsorption (38). Previous studies have found that neurological function and imaging findings at admission have significant predictive value. A meta-analysis showed that the NIHSS score within 6 h of admission is a reliable predictor of MCE (39). Thomalla et al.’s study established an NIHSS score ≥18 as a predictive threshold with good predictive performance (40). Similarly, an ASPECTS score ≤7 not only suggests a larger core infarct volume but also predicts a higher risk of cerebral edema (41). Regarding the impact of vessel recanalization, multiple studies have consistently shown that successful recanalization is significantly associated with a reduced risk of MCE (42, 43). Kimberly et al.’s study found that recanalization can reduce the incidence of midline shift (6), a finding further confirmed in a subsequent systematic review that included 38 studies (26). This protective effect may be related to improving cerebral tissue perfusion in the ischemic penumbra and reducing edema-related brain injury. Interestingly, we observed that smoking history was associated with a reduced risk of MCE. Although the protective mechanism has not been fully elucidated, evidence suggests that nicotine may exert neuroprotective effects by activating the endogenous cannabinoid system (44). Nicotine promotes the release of endogenous cannabinoids and induces mild hypothermia, thereby inhibiting inflammatory responses and alleviating cerebral edema (45).

Although the AUC values for liver fibrosis indices in our study were below 0.70, indicating moderate predictive performance, these findings hold significant clinical value and research implications. First, malignant cerebral edema represents a complex, multifactorial pathophysiological process where single biomarkers rarely achieve perfect predictive performance. Second, liver fibrosis indices serve as novel predictive factors that provide valuable complementary information to existing prediction models. In the era of precision medicine driven by multimodal medical data, integrating multiple biomarkers to construct comprehensive prediction models has become standard practice. Liver fibrosis indices can be combined with established clinical scoring systems (such as NIHSS and ASPECTS scores) and other biomarkers to enhance overall predictive efficacy. Notably, significant associations between liver fibrosis indices and MCE risk were observed despite normal AST, ALT, and platelet values in both groups. This finding enhances the clinical relevance of our results by demonstrating that composite fibrosis indices can detect subclinical hepatic dysfunction before individual parameters become abnormal.

Stroke patients with elevated liver fibrosis indices may benefit from multilevel therapeutic strategies. Etiology-directed treatments include antiviral therapy for chronic viral hepatitis to effectively suppress viral replication, relief of cholestasis or treatment of underlying causes, weight management and metabolic disorder correction for non-alcoholic fatty liver disease patients, and strict alcohol cessation for alcoholic liver disease patients. These measures can reduce ongoing hepatic injury and promote fibrotic tissue repair. Lifestyle interventions include dietary modifications, regular exercise, and metabolic syndrome control. Integrating liver fibrosis management into comprehensive stroke care has multiple implications: first, improving hepatic health may reduce malignant cerebral edema risk through mechanisms including reduced systemic inflammation and improved endothelial function; second, this comprehensive management strategy may decrease long-term all-cause mortality and prevent stroke recurrence; finally, dynamic monitoring of liver fibrosis indices provides clinicians with valuable information regarding disease progression and treatment response, guiding personalized therapeutic decision-making adjustments.

This study has several limitations that need to be addressed. As a single-center retrospective cohort study, the results may be subject to selection bias, and external validity needs to be verified. Although multiple validated non-invasive liver fibrosis indices were used, the lack of liver biopsy as the gold standard may affect the accuracy of liver fibrosis assessment. Additionally, all liver fibrosis indices investigated in this study involve complex mathematical calculations requiring multiple laboratory parameters, which may limit their practical implementation in daily clinical practice and potentially lead to calculation errors when performed manually. There is a need to develop user-friendly digital applications or automated calculation tools integrated with electronic health record systems to facilitate real-time risk assessment and improve clinical workflow efficiency. Furthermore, although this study confirmed the association between liver fibrosis and MCE after EVT, specific intervention strategies have not been elucidated. To address these limitations, future research should focus on the following aspects: First, conduct multicenter prospective cohort studies, integrate imaging examinations such as liver elastography, and establish a more accurate liver fibrosis assessment system; second, explore the molecular mechanisms by which liver fibrosis promotes the occurrence and development of MCE, and verify whether potential existing hepatoprotective drugs have neuroprotective effects; finally, conduct individualized treatment studies based on liver fibrosis stratification and develop standardized monitoring and intervention protocols. These studies will contribute to a better understanding of the role of liver fibrosis in stroke prognosis and provide new strategies for improving the efficacy of EVT treatment.

## 5 Conclusion

Non-invasive liver fibrosis indices serve as novel predictive biomarkers for malignant cerebral edema following endovascular therapy in anterior circulation large vessel occlusion stroke. Elevated FIB-4, modified FIB-4, AST/ALT ratio, and AARPRI were independently associated with increased risk of malignant cerebral edema, while FIB-5 showed a negative association. These readily available clinical tools may help identify high-risk patients and guide early preventive interventions, potentially improving clinical outcomes after endovascular therapy.

## Data Availability

The raw data supporting the conclusions of this article will be made available by the authors, without undue reservation.
